# Allopathic Progress

**Published:** 1896-02

**Authors:** Edmund J. Lee

**Affiliations:** Philadelphia


### ALLOPATHIC PROGRESS.

Edmund J. Lee, M. D., Philadelphia.

We move so fast in these days that it is well to pause occasionally to take our bearings, to see just where we are, and to test our progress to see if it be genuine. We of the homœopathic school, guided by an unerring law of nature, are in little danger of wandering from the true path; if we are truly guided in all our studies of drugs by that law, our course will always be a continued advance, never a zigzag or retrograde one, a frequent result of mere experimentation.

The allopathic school, on the other hand, being ruled by no law of nature in its study and use of drugs, is generally guided solely by the fad of the moment. To-day this fad is the study of antitoxic remedies; a truer name for them would be isopathic, for such they are. These preparations are really isopathic in their purpose, and differ from those sometimes used in our school by their mode of preparation and administration. With all due respect to the scientific gentlemen of the old school who are now investigating this class of remedies, it may be safely asserted that dilution by means of pure alcohol is more scientific and healthier than "cultivation" by means of animals. In what respect does "antitoxin" differ from "diphtherinum" in preparation and use? One is diluted by "cultivation;" the other by alcohol. One is used in poisonous doses to overcome, crush out, the diphtheritic disease; the other is given only to such cases as its pathogenesis demands. Which method is the more scientific, the more accurate, and the less dangerous?

Many remarkable discoveries are being made in the old school. "It is only," says a distinguished member of that school, "during the last decade that the unity of the human body and the consequent influence of general conditions upon local ones has been adequately appreciated. * * * Modern times have witnessed the establishment of the valuable thought that chronic lesions are but physical expressions which tell of a greater or less depression of the general vitality." We have long been under the impression that Hahnemann in his *Organon* had said something of this connection between local and constitutional diseases. But perhaps this writer was graduated from one of our (so-called) homœopathic colleges, and hence is entirely excusable for not knowing anything of the contents of *The Organon*.

Among the many new remedies introduced recently to the medical profession by their kind friends, the manufacturing chemists, are preparations made of different organs of the body, such as thyroid and thymus glands, the spleen, spinal cord, brain, heart, etc. Of the value of these preparations, we cannot give an opinion, but must say that the treatment of goitre by internal administration of "desiccated thyroids" seems closely akin to isopathy.

The use of parts of the body in medicine is probably as old as medicine itself. Certainly the old works on medicine teemed with such articles, and it may be interesting to quote some of these from an old medical work to show the progress of our allopathic friends. It would seem that their progress follows a curved rather than a straight line!

In 1678 William Salmon, "professor of Physick," translated into English "for the publick Good," the *New London Dispensatory*. In it many curious formulæ are given; for want of space only a few of them can be quoted here. Many parts of the human body are recommended for various disorders. For instance, the hair; the "Powder thereof drunk cures the Jaundice and suffocation of the Womb: the Ashes of it mixt with Hogs-lard, and annointed helps luxated Joynts; the Ashes stop bleeding."

The nails: "in powder or infusion, cause Vomiting, great sickness at Stomach and giddiness in the Head; the Powder laid to the Navel in Dropsies is said to cure them." "Calculus, Stone taken from the Kidneys or Bladder. It dissolves and expels the Stone and Gravel from all parts and opens Obstructions being given ʒj at a time in powder." "Balsamum Arthriticum" was made of "Mans blood putrified ten days and distilled from sand by degrees," etc.; it had "Strange force in Gout." Mummies in various forms were the sheet-anchors of

Professor Salmon; he names five varieties, and laments that 'the Arabian is scarcely to be got; the second and third [varieties] are sold for it." Showing that even in ye olden time druggists occasionally were unreliable. "Mumia Artificialis, Artificial or Modern Mummy," according to Crollius, are thus made: "Take the Carcass of a young man (some say red hair'd) not dying of Disease, but killed; let it lie 24 hours in clear water in Air: cut the flesh in pieces, to which add powder of Myrrh, and a little Aloes: imbibe it 24 hours in the spirits of Wine and Turpentine, take it out, hang it up twelve hours; imbibe it again in fresh spirit, then hang up the pieces in a dry air, and shadowy place, so will they dry and not stink. * * * It is a counter-poyson, prevents the Plague, and resists all manner of Infection, being taken only to ℈j, and cures being taken to ʒj or ʒji."

Human bones stopped "fluxes of the Belly, of Rheum, of the Terms, astringe and take pains of the gout." The skull "is a specific in the cure of most Diseases of Head but chiefly the Falling Sickness; you may give it either levigated on a Marble, or calcined or some of the following preparations thereof; the Triangular Bone in the Temple is the most Specifical against Epilepsie." Professor Salmon did not approve of Paracelsus' "Extract of Man's Skull;" "it is much to be questioned," he wrote, "whether it does not carry away with it much of the finer spirit of the skull." Aqua et Oleum Cranii humani was "one of the most powerful things against Falling Sickness and suffocation of the Womb."

Spiritus Cerebri Humani was "a noble Antepileptick," and was prepared in this manner:

"℞ The Brain of a young man slain, with all its membranes, Arteries, Veins, and Nerves, with all the Spinal Marrow, beat them, and add Essence of Tile-flowers, Peony, Bettony, black cherries, Lavender, Rosemary, Lilly of the Vally, Cowslips, Sage, Mestleto, ana, so much as to be four inches above; digest a while; then distil in B. M. add Sack a fourth part, distil and cohobate three times; make a salt of the feces calcin'd, which joyn to the spirit."

"Fel Humanum, Mans Gall," says the professor, "dropt into the Ear cures deafness;" and the "Cor hominis, The Heart, The Powder of it drunk cures the Epilepsie."

In addition to parts of the human body, those of all manner of beasts, fishes, birds, and reptiles were used; only a few instances can be quoted here, and it is curious to note the use of organs and secretions to cure diseases of those parts. Thus, urine was often given for strangury, stone in the bladder, etc.; blood "stops and heals all manner of fluxes, stops spitting of blood," etc. The liver of the cow "strengthens the liver in man;" the broth of the spleen "cures hard spleens, and provokes the Terms."

Of the toad, Bufo, this statement is given: "Scroder saith, That there is such a great antipathy between a Toad and a Spider; that if a Spider be put upon a Toad, it will burst the Toad violently. They are hung up by the neck in the Air, till they are through dry, and then kept for use. Wierus saith, That the Powder of a dry'd Toad, taken ʒ fs at a time, or more, cures almost incurable Dropsies, carrying away the water by Urine; I suppose the ashes of them burnt is better. A dried Toad stept in Vinegar, and the belly of it laid to a Carbuncle, draws out the Poyson; smelt to it stops bleeding at nose, especially laid to the forehead, or behind the Ears, or held in the hand till it is hot, or hung about the Neck. The Ashes or Powder do the same, laid upon the part that bleeds. Laid to the Reins, it cures the Dropsie; to the Navil, Fits of the Mother; Laid to the soles of the feet, it helps distempers of the Head and Heart; and cools the heat of Feavers. The Ashes hung about the Neck (as an Amulet) cures pissing a bed, or the not holding of the Water."

Of the honey bee we are told that "the whole Bee in Powder given inwardly, provoke Urine, opens all stoppages of the Reines, and break stone; they are good against Cancers, Schirrous Tumors, the Kings Evil, Dropsy, dimness of sight, for being taken a good while, they wast the Humor, and restore health."

In these comments on Bufo and Apis there is a glimpse of their curative properties which homœopathic pathogeneses have fully revealed. Perhaps provings might also bring to light valuable curative power in the various animal products recently introduced to the notice of the old-school doctors.

